# An Interdisciplinary Diagnostic Approach to Guide Therapy in C3 Glomerulopathy

**DOI:** 10.3389/fimmu.2022.826513

**Published:** 2022-05-27

**Authors:** Tilman Schmidt, Sara Afonso, Luce Perie, Karin Heidenreich, Sonia Wulf, Christian F. Krebs, Peter F. Zipfel, Thorsten Wiech

**Affiliations:** ^1^ III. Department of Medicine, University Medical Center Hamburg-Eppendorf, Hamburg, Germany; ^2^ Department of Infection Biology, Leibniz Institute for Natural Product Research and Infection Biology, Hans-Knöll-Institute, Jena, Germany; ^3^ Medical Alliance, eleva GmbH, Freiburg, Germany; ^4^ Nephropathology Section, Institute of Pathology, University Hospital Hamburg Eppendorf, Hamburg, Germany; ^5^ Division of Translational Immunology, III. Department of Medicine, University Medical Center Hamburg-Eppendorf, Hamburg, Germany; ^6^ Hamburg Center for Translational Immunology (HCTI), University Medical Center Hamburg-Eppendorf, Hamburg, Germany; ^7^ Institute of Microbiology, Friedrich Schiller University, Jena, Germany

**Keywords:** C3 glomerulopathy, membranoproliferative glomerulonephritis, complement, factor H, eculizumab, FHL1

## Abstract

Since the re-classification of membranoproliferative glomerulonephritis the new disease entity C3 glomerulopathy is diagnosed if C3 deposition is clearly dominant over immunoglobulins in immunohistochemistry or immunofluorescence. Although this new definition is more orientated at the pathophysiology as mediated by activity of the alternative complement pathway C3 glomerulopathy remains a heterogenous group of disorders. Genetic or autoimmune causes are associated in several but not in all patients with this disease. However, prognosis is poorly predictable, and clinicians cannot directly identify patients that might benefit from therapy. Moreover, therapy may range from supportive care alone, unspecific immune suppression, plasma treatment, or plasma exchange to complement inhibition. The current biopsy based diagnostic approaches sometimes combined with complement profiling are not sufficient to guide clinicians neither (i) whether to treat an individual patient, nor (ii) to choose the best therapy. With this perspective, we propose an interdisciplinary diagnostic approach, including detailed analysis of the kidney biopsy for morphological alterations and immunohistochemical staining, for genetic analyses of complement genes, complement activation patterning in plasma, and furthermore for applying novel approaches for convertase typing and complement profiling directly in renal tissue. Such a combined diagnostic approach was used here for a 42-year-old female patient with a novel mutation in the Factor H gene, C3 glomerulopathy and signs of chronic endothelial damage. We present here an approach that might in future help to guide therapy of renal diseases with relevant complement activation, especially since diverse new anti-complement agents are under clinical investigation.

## Introduction

In 2013 a consensus report suggested a re-definition of the heterogeneous group of membranoproliferative glomerulonephritis (MPGN) (1, 2). A new classification according to the immunohistochemical/immunofluorescence findings was recommended, in order to allow better association of pathogenesis of the diseases compared to the pure morphological distinction of MPGN type I, II, or III. For that, C3 glomerulopathy (C3G) should be diagnosed if C3 deposition is clearly dominant over immunoglobulins. This new definition of C3G includes the patterns MPGN I and III, as well as intramembranous glomerulonephritis/dense deposit disease (MPGN type II). Moreover, diagnosis of C3G was not restricted to a membranoproliferative pattern but could be every other form of glomerulonephritis, e.g., mesangioproliferative. This new definition resulted from advances in the understanding of complement-mediated kidney diseases, of which C3G is one prototypical disease (3, 4). In C3G, overactivation of the complement system can be associated with genetic mutations in complement genes, like Factor H, C3 and the *FHR1*, *FHR2*, *FHR3*, *FHR4* and *FHR5* genes. Nephritic factors are antibodies that are capable to stabilize complement activation by binding to the alternative pathway (AP) C3 convertase or the C5 convertase or to single complement proteins such as Factor H, C3, C3b, C3d or Factor B. These diverse antibodies interfere with the alternative pathway activation and cause its overactivation. In healthy individuals the alternative complement pathway is constantly activated by default, due to a spontaneous hydrolysis of C3 and controlled by different complement factors. Complement factor H (Factor H) is the primary regulator of the alternative complement cascade. The Factor H genes encode two mRNAs. One codes for the full length Factor H gene which is composed of 20 repeat domains The second mRNA encodes FHL1 a 42 kDa plasma protein that includes the first seven SCR domains of Factor H. Several other mutations, primarily interacting with the alternative complement pathway, have been described. Mutations in other genes link in FHR1, FHR2, FHR3, FHR4 FHR5, C3 included genes which encode components that form the C3 or C5 convertases or for regulators which define the time and the site of C3 convertase action (5).

However, in a relevant number of patients, a causal genetic alteration or autoimmune factor cannot be found. Despite the new classification and orientation towards pathophysiology, C3G remains a very heterogeneous disease. In consequence, the clinical outcome of the patients is different. While some patients initially present with rapidly progressive glomerulonephritis, others present with albuminuria and have stable renal function. The mean 10-year renal survival rate is approximately 50% (6). Clinicians first have to decide whether or not to treat a C3G patient, but there are only few studies focusing on the therapy of this rare disease group. Some of the studies were performed before reclassification, meaning that these studies do not only include C3G but also immune complex forms. Given the change in terminology and disease characterization and the potential confounding effect on trial stratification, the results of these trials are of limited use in guiding current treatment considerations for C3G. In general, there are different treatment strategies, including immunosuppression, plasma therapy, or complement blockade. A significant dilemma is that clinicians lack data on which therapy might be helpful in which patients. The current diagnostic work-up seems not sufficient to guide treatment of single patients. We want to highlight the importance of an interdisciplinary diagnostic approach to understand an individual patient’s form of C3G to guide therapies with this work.

## Case Analysis

**Figure 1:**
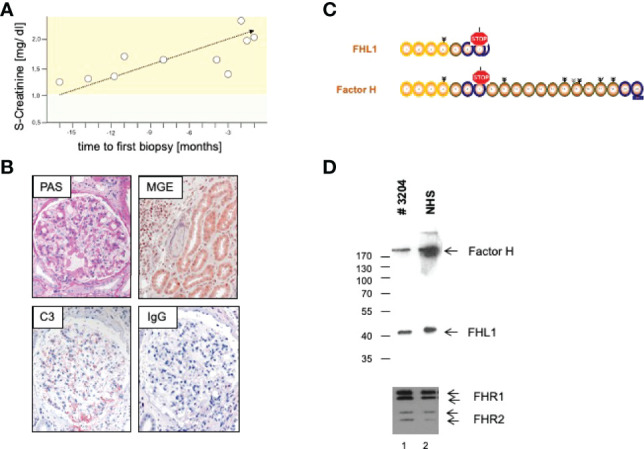
**(A)** Course of S-Creatinine values previous to treatment. **(B)** Representative microphotographs of periodic acid Schiff (PAS) and Masson-Goldner-Elastica (MGE) stained kidney sections show a mesangioproliferative pattern (PAS) and signs of chronic endothelial damage in an arteriole (MGE). Immunohistochemical staining reveal strong positivity for C3, but no deposition of Immunoglobulin G (IgG). **(C)** Genetic analysis revealed introduction of a stop codon in domain 7 with a **(D)** consecutive a lack of Complement Factor H (Factor H) and Factor H like Protein1 (FHL1) in serum of the patient.

**Figure 2:**
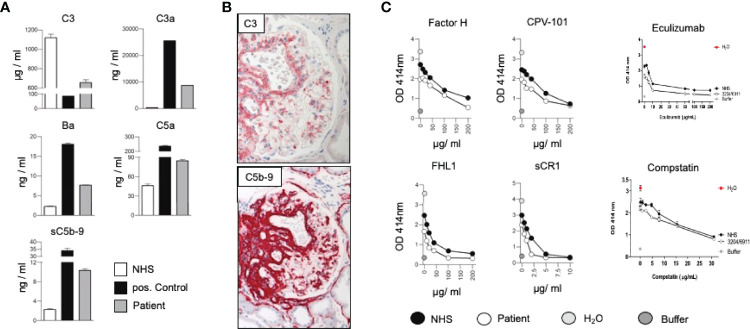
**(A)** Measurement of the complement protein 3 (C3), the cleavage product C3a, Factor Ba, fragment, complement anaphylatoxin C5a, and the soluble form of the membrane attack complex (sC5b-9) in sera of the patient, a healthy subject (NHS) and a patient suffering from hemolytic uremic syndrome induced by deficiency of complement Factor H (positive control) by ELISA. By following the levels of C3, the central complement components indicative of continuous ongoing complement activation at the early levels (8). The consumption of C3 combined with elevated levels of C3a, Ba, C5a and sC5b-9 shows that complement is activated and that activation proceeds to the C3 convertase, as well as C5 convertase level and processes to the terminal pathway. Such strong fluid phase activation is in agreement with reduced Factor H and FHL1 plasma levels. **(B)** Representative immunohistochemical staining for C3 and the membrane attack complex (C5b-9). **(C)** *In vitro* effect of Factor H, CPV-101, FHL1, sCR1 Eculizumab and Compstatin in *in vitro* hemolysis assays using patient serum (white circles) NHS (black circles), H2O (grey circles) or buffer (dark grey circles).

We here present a case of a 42-year-old female, Caucasian patient that presented with arterial hypertension, elevated serum creatinine values and albuminuria (up to 500 mg/g creatinine) at her nephrologist. Due to ongoing rise of serum creatinine the patient was sent to our hospital (**Figure 1A**). A kidney biopsy was performed, revealing the diagnosis of mesangioproliferative glomerulonephritis. Immunohistochemical staining for immunoglobulins A and G was negative. In contrast, there was dominant mesangial positivity for C3 (**Figure 1B**), leading to the diagnosis of a mesangioproliferative glomerulonephritis with dominant C3 deposition. Since there was no evidence for an infection-related cause, it was classified as mesangioproliferative C3 glomerulonephritis as a subform of C3G. Due to the ongoing impairment of renal function, we agreed that there was an indication for treatment. Since clinical trials are not sufficient to recommend a specific therapy for these cases, we performed further analysis. Genetic testing revealed a heterozygous mutation of the Factor H gene, introducing a stop codon at p.Pro440 -Stop, in SCR7 (**Figure 1C**). Such heterozygous Factor H mutations in domain 7 are not described so far for C3G and to our knowledge most mutations in the Factor H gene associated with C3G or MPGN type II present as homozygous or compound heterozygous settings. The stop codon in domain 7 affects expression of one allele both of Factor H and of the FHL1 protein. Indeed, plasma protein levels of both Factor H and FHL1 were low when compared to healthy subjects (**Figure 1D**). Interestingly, other mutations of Factor H are associated with thrombotic microangiopathies, especially atypical hemolytic uremic syndrome (7). Indeed, the initial biopsy showed some intimal sclerosis without elastosis (**Figure 1B**), which could be regarded as a former or chronic thrombotic microangiopathy, indicating an ongoing damage of endothelial cells. However, the biopsy did not reveal fresh microthrombi. Nevertheless, the genetic analysis linked glomerulonephritis and the (alternative) complement system and so strengthened the diagnosis of C3 glomerulonephritis. To evaluate different therapeutic strategies, we performed further analyses. Using ELISA, a series of complement markers and activation product were analyzed in plasma to follow the complete complement pathways and compared their pattern with a patient with atypical hemolytic uremic syndrome as with other patients and also healthy subjects (**Figure 2A**) (8). C3 consumption combined with increased levels of complement activation products indicates ongoing complement activation, which is also detected in plasma of a patient with genetically mediated atypical hemolytic uremic syndrome. C3 level were reduced in patient serum. In addition C3a, a product of C3 hydrolysis and opsonin, was increased when compared with healthy subject. Moreover, the noncatalytic subunit Ba of complement Factor B was upregulated considerably, further indicating activation of the alternative complement pathway. Since the patient suffered from a mutation that could not control complement overactivation, we assumed activation downstream of the C3 convertase, e.g., the terminal complement pathway. Indeed, we detected the terminal complement cascade products to be elevated (C5a and C5b-9) (**Figure 2A**). The observation of terminal complement activation was not restricted to the serum reflecting defective fluid phase regulation. Analyzing the kidney biopsy, prominent immunohistochemical staining for C3 and, even more impressive, C5b-9 we observed (**Figure 2B**) arguing for activation of the terminal complement pathway also on the surface of the target organ.

**Figure 3:**
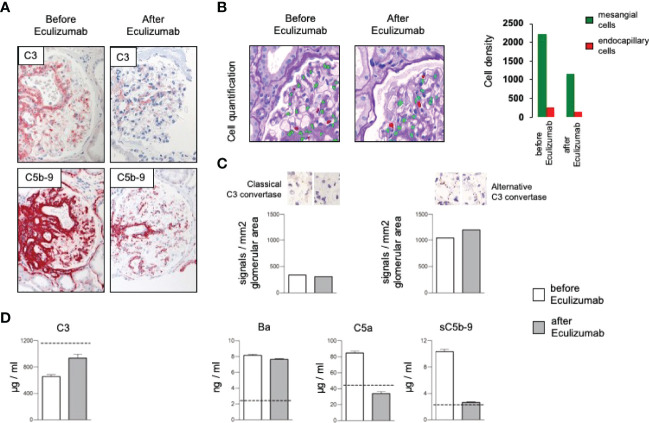
**(A)** Representative immunohistochemical staining for complement C3 with a mild and the membrane attack complex (C5b-9) with a significant reduction after treatment with Eculizumab. **(B)** Analyzing density of mesangial and endocapillary mononuclear cells shows a reduction of both cell types. **(C)** Quantification of the signal density for the classical and the alternative C3 convertase revealed no major changes, especially no reduction of the alternative convertase. **(D)** Complement marker profile before and after Eculizumab treatment. C3 plasma levels increased upon Eculizumab therapy. The proximal activation markers C3a, and Ba did not change. Distal activation markers, e.g. the anaphylatoxin C5a and the soluble membrane attack complex (sC5b-9) showed reduction after treatment with Eculizumab.

Due to complement activation with the apparent involvement of the terminal cascade, signs of thrombotic microangiopathy, and the Factor H/FHL1 mutation, we asked which type of therapy might be best for this patient. Assuming that reduced plasma levels and reduced regulatory Factor H and FHL1 function caused complement deregulation in plasma and on surfaces in the patient, we wanted to test which components, Factor H, FHL1 or complement inhibitors can restore the complement stability and reduce hemolysis. To this end we established *in vitro* hemolytic assays to the show that serum purified Factor H, CPV-101 H, FHL1, Compstatin. sCR1 or also Eculizumab might influence complement mediated hemolysis of sheep erythrocytes, and further more asking whether complement preferred C5 inhibition as the most promising treatment strategy. Both, Factor H, CPV-101, FHL1 and sCR1 reduced hemolysis in this assay and the effects were comparable and dose dependent. The C3 inhibitor (Compstatin) also reduced hemolysis. Moreover, Eculizumab, at that time the only clinically approved inhibitor, also inhibited lyses (**Figure 2C**). Based on these effects we finally decided to start treatment with Eculizumab. After 13 weeks of treatment, again a kidney biopsy was performed, allowing us to reevaluate the inhibitory effect in the target organ. Upon treatment we observed some reduction of C3 and considerable reduction of C5b-9 deposition in the glomeruli (**Figure 3A**). Using a computer-based deep-learning assisted detection of cell nuclei, we quantified mesangial and endocapillary mononuclear cells (**Figure 3B**). With that tool, we found a reduction of both cell types, indicating functional relevance of reduced C5a and C5b-9 deposits. As previously described, we are also able to visualize the alternative and classical C3 convertase using proximity ligation assay (PLA) (**Figure 3C**) (9). Both were unaffected by the C5 blockade, assumably since Eculizumab blocks downstream of the C3 convertases. In line with this argumentation, serum analysis revealed a dramatic reduction in the terminal complement components (C5a, C5b-9). Components upstream of C5 inhibition, e.g. C3 and Ba, were not affected (**Figure 3D**). By including these PLA methods, theoretically it is also possible to distinguish whether the reduction of C5b-9 deposition is a therapy effect or reflects a decreased natural disease activity. In this case, unchanged high signal numbers for the alternative C3 convertase indicates an unchanged natural proximal disease activity, so that the C5b-9 reduction can be interpreted as a therapy effect. However, also C3a was reduced upon Eculizumab treatment, potentially arguing for a feedback regulation on C3 conversion. During treatment the serum creatinine levels stabilized (1.5 – 1.7mg/dl) and the patient presented without significant albuminuria (< 100mg/g creatinine), up to 20 weeks follow-up.

## Discussion

C3G is a heterogeneous disease group resulting from defective complement regulation either in the plasma (fluid phase) or on surfaces such as the glomerular basement and cell membranes. Different mechanisms ultimately drive the same pathological principals. The disease ranges from genetic mutations to autoantibody-mediated dysregulation of the alternative complement system. In dense deposit disease, 25 of 32 patients (78%) were positive for an autoantibody stabilizing the alternative C3 convertase (10). Genetic analysis demonstrated several mutations in various genes interacting with alternative complement cascade, including Factor H, FHR1, FHR2, FHR3, FHR4, FHR5 and C3 (1, 3). However, immune-complex mediated MPGN and C3G share pathophysiological aspects and even immunoglobulin dominant MPGN cases can have an underlying, primary dysregulation of the alternative complement pathway (6, 11–13). Thus, also in these cases, if systemic infectious or rheumatological diseases, such as systemic lupus erythematosus, hepatitis C, and cryoglobulinemia are excluded, it is important to consider autoimmune, genetic, and functional plasma analysis for the alternative pathway. The clinical course of C3G is difficult to predict. The Mayo Clinic published its data on 114 C3G patients and concluded variable response to their therapy, meaning that there is no clear data on when therapy is indicated in an individual patient (14). Currently, a kidney biopsy is mostly performed at the initial stage of disease and is primarily of diagnostic relevance. However, endocapillary hypercellularity or necrosis/extracapillary proliferation may serve as criteria for treatment. Up to now, authors recommend unspecific therapy with steroids or the combination with proliferation inhibitors (15). Guidelines and most authors recommend MMF as the first-line therapy and refer to one study if immunosuppression is indicated. In a Spanish cohort, MMF in combination with steroids demonstrated preferable result when 22 patients were compared to 18 patients that received other immunosuppression (mostly steroids alone) (16). This observation was confirmed in another small study in which 67% of 30 patients treated with MMF were classified as responders to immunosuppression (17). The largest study investigating the therapeutic effect of MMF in C3 glomerulopathy includes 97 patients (C3G n=81; DDD n=16). Here, not only the therapeutic response of MMF and steroids was investigated in comparison to other therapeutic options, but also the different pathogenesis with regard to genetic alterations and antibodies was taken into account. After a follow-up of up to 10 years, a superiority of the group treated with MMF and steroids was shown. This was true for the comparison with other immunosuppressive therapy regimens as well as for supportive therapy alone. However, the therapeutic effect of MMF and steroids seemed to be less pronounced in patients with genetic abnormalities (18). These observations contrasts with other reports which demonstrate no benefit for immunosuppression (19, 20). Given the heterogenous character of the disease some cases of complement-mediated glomerulonephritis seem to benefit from immunosuppression. Such forms might represent the former with complement activation in plasma as indicated by reduced C3 plasma levels as well as elevated levels of the inflammatory anaphylatoxins C3a or C5a. Kidney biopsy from such patients may lack signs of thrombotic microangiopathy, but might show glomerulonephritis with an influx of inflammatory cells and mesangial cell proliferation. This case underlines the importance of establishing multiple complement plasma markers upon time of diagnosis and combine this with genetic testing. We firstly demonstrate a new heterozygous Factor H gene mutation associated with C3G, which affects both Factor H and FHL1 levels in plasma. Since Factor H and FHL1 are strong inhibitors of alternative complement, reduced levels Factor H and FHL1 might force clinicians to treat patients with plasma infusions or exchange. Our analysis gained insights into the therapeutic effect after reconstitution with Factor H and FHL1 by *in-vitro* testing. Indeed, this may be a relevant therapeutic option. Licht et al. also demonstrated the efficacy of plasma infusions in two patients suffering from DDD due to a Factor H mutation and preserved them from disease progression (21). However, even if treatment was well tolerated in this study, plasma infusion risks allergic reactions and might cause volume overload. Moreover, as C3G potentially progresses to end-stage kidney disease, plasma infusion can cause antibody formation, which must be avoided before renal transplantation. Supplementation of Factor H by recombinant proteins might be a therapeutic prospect. Its production in therapeutically useful quantities might be feasible by production in several cellular systems (22). Restoring Factor H activity by human recombinant Factor H in deficient knockout mice led to resolution of glomerular basement membrane lesions in a murine model for dense deposit disease (23). This might be a therapeutic option for patients with complement mediated disease suffering from loss of function Factor H mutations.

The new approach presented here shows that a detailed analysis of the kidney biopsy in combination with extensive complement marker analysis in plasma is helpful at the time of diagnosis, allows to evaluate complement inhibitors for therapy and furthermore allows to follow the complement response upon treatment with complement inhibitors. In addition, repeated biopsies following therapy with complement inhibitors show the effect of treatment in the affected organ, and these effects were combined with inhibition of the activation in plasma, maybe allowing to monitor or even predict clinical response. We detected low levels of C3 and consecutive higher levels of C3a, indicating complement activation. Ba fragment is cleaved from Factor B upon formation of the alternative C3 convertase (C3bBb). Elevated Ba levels strongly argue for alternative activation in plasma since Factor B is only involved in this pathway. A detailed examination of combined complement markers along the cascade allows to follow the complement activation steps and this may help to narrow down the localization of the defect leading to extended complement activation. Given different cause single patients might present with a different pattern of complement activation. With such a detailed analysis of complement components, it might be feasible to choose the right drug for an individual patient. In our case, elevated C5b-9 levels in serum and the kidney biopsy argued for a dominant role of the terminal complement cascade. Despite several successful case reports on Eculizumab in C3G, this treatment seems to have mixed results (24, 25). In a study by Bomback, with six patients (three DDD, three C3G) that presented with albuminuria >1g/d and/or AKI were treated for 12 months (26). During that course of treatment, only three patients had a marked reduction of s-creatinine or albuminuria and one more patient had histopathological evidence of improvement. In the French cohort in a heterogeneous group of 26 patients, including 13 adolescents and 13 children, some of them having CKD, others presenting with progressive disease, and at least two patients requiring dialysis were treated for a median duration of 14 months (27). 54% of these patients had no clinical response. In study of 10 patients that presented with nephrotic albuminuria Eculizumab was used in a single-arm trial in an off-on-off-on design. Patients were treated in two 48-week treatment periods, divided by a 12 week washout period. In this study, only patients with very high levels of sC5b-9 levels were included. Despite this evidence for activation of the terminal complement cascade, only 30% of the patients achieved significant reduction of proteinuria in the first treatment period. All responders showed increase of proteinuria during eculizumab withdrawal (28). However, none of these studies were able to predict the response to the treatment. In this case, we were convinced that C5 inhibition might be the favorable treatment strategy since we found overactivation of the alternative complement pathway and a genetic mutation that cannot control that overactivation. Consequently, we saw the activation of the terminal complement pathway in our patient’s sera and kidney. In addition to C3G, the renal biopsy had also shown signs of an old thrombotic microangiopathy, which can occur in atypical hemolytic uremic syndrome. Mutations in Factor H are also present in aHUS, in which eculizumab is an approved therapy. Indeed, the efficacy of eculizumab has been demonstrated in 2 patients with bioptic signs of C3G and thrombotic microangiopathy (29). After *in-vitro* testing the therapeutic potential, we started treatment with Eculizumab. Even if the follow-up period is too short to draw conclusions about the effective efficacy of eculizumab treatment we detected an inhibition of terminal complement activation in the sera and the kidney. Since Eculizumab acts downstream of the C3 convertase, changes in the concentration of C3a and Ba were less affected under C5 inhibition. However there are signs of a feedback regulation under Eculizumab. Since current recommendations focus on classical risk factors for progressive kidney diseases and do not consider genetic and functional analysis we argue that a combined and detailed analysis of the plasma complement parameters and the kidney might allow guiding therapy in C3G and also in other complement-mediated glomerulonephritis. A more detailed insight into different (alternative) complement activation steps might be more relevant soon since various complement blocking agents are under clinical investigation for renal and extrarenal diseases (30).

In conclusion, we believe that C3G is not only too rare but also too heterogenous for larger, controlled, randomized, prospective therapy studies. Thus, C3G is a perfect example of a disease, in which unravelling the exact pathogenesis by combining morphological *in situ*, genetic, autoimmune, and functional *in vitro* data of single patients likely will be the clue to the best, personalized therapy.

## Data Availability Statement

The original contributions presented in the study are included in the article/supplementary material. Further inquiries can be directed to the corresponding author.

## Ethics Statement

The patients/participants provided their written informed consent to participate in this study. Written informed consent was obtained from the individual(s) for the publication of any potentially identifiable images or data included in this article. The patient’s consent is existent *via* the Hamburg Glomerulonephritis Registry approved by the Hamburg Ethics Committee (approval number PV4806).

## Author Contributions

SA, LP, KH, and PFZ performed functional analyses; CK also contributed to manuscript preparation. CK provided clinical data; SW, TS, and TW performed morphological and *in situ* analyses; TS, PFZ, and TW wrote the manuscript. All authors contributed to the article and approved the submitted version.

## Funding

This work was supported by a grant from the Deutsche Forschungsgemeinschaft as part of the collaborative research program ‘Immune-Mediated Glomerular Diseases’ SFB1192, Project B6 to TW and PFZ, Project C1 to TW, as well as the Kidneeds foundation to PFZ.

## Conflict of Interest

Author KH is employed by eleva GmbH. TS received advisory fees from Alexion. PFZ received consulting fees from eleva GmbH. This support did not influence the research work or the content of this manuscript. TW and PFZ have received speaker fees from Bayer and Novartis, TW has received speaker fees from GlaxoSmithKline GmbH. TW, SW and PFZ are authors of a patent application for the detection of complement convertases in tissue.

The remaining authors declare that the research was conducted in the absence of any commercial or financial relationships that could be construed as a potential conflict of interest.

## Publisher’s Note

All claims expressed in this article are solely those of the authors and do not necessarily represent those of their affiliated organizations, or those of the publisher, the editors and the reviewers. Any product that may be evaluated in this article, or claim that may be made by its manufacturer, is not guaranteed or endorsed by the publisher.
